# EFFICACY OF A NEW VIDEO OBSERVATIONAL TRAINING METHOD (INTENSIVE VISUAL SIMULATION) FOR MOTOR RECOVERY IN THE UPPER LIMB IN SUBACUTE STROKE: A FEASIBILITY AND PROOF-OF-CONCEPT STUDY

**DOI:** 10.2340/jrm.v56.36119

**Published:** 2024-09-25

**Authors:** Etienne OJARDIAS, Ahmed ADHAM, Hugo BESSAGUET, Virginie PHANER, Diana RIMAUD, Pascal GIRAUX

**Affiliations:** 1 Physical Medicine & Rehabilitation Department, University Hospital of Saint-Étienne, Saint-Étienne, France; 2 Lyon Neuroscience Research Center, Trajectoires team (Inserm UMR-S 1028, CNRS UMR 5292, Lyon1 & Saint-Etienne Universities), France; 3 Inter-University Laboratory of Human Movement Biology, EA 7424, Jean Monnet University, Saint-Etienne, France

**Keywords:** equipment and supplies, evaluation study, feedback, sensory, hemiplegia, stroke rehabilitation, upper extremity, intensive visual simulation, IVS

## Abstract

**Objective:**

To demonstrate the feasibility and efficacy of a new video-observation training method (intensive visual simulation) to improve upper limb function.

**Design:**

Small sample, randomized, evaluator-blind, monocentric study.

**Patients:**

Seventeen early subacute ischaemic stroke patients with complete hemiplegia were randomly assigned to the therapeutic group (*n* = 8) or control group (CG, *n* = 9).

**Methods:**

Thirty sessions of intensive visual simulation combined with corrected visual feedback (therapeutic group) or uncorrected visual feedback (control group) were performed over 6 weeks on top of a standard rehabilitation programme. Main outcome measure: 400-point hand assessment test (400p-HA). Secondary outcome measures: Box and Blocks (B&B), Purdue Pegboard test, Minnesota.

**Results:**

The 400p-HA test improved significantly from T0 to 6 months for both groups, with a significant difference between groups at 3 months (MW-UT *p* = 0.046) and 4 months (MW-UT *p* = 0.046) in favour of the therapeutic group. One-phase exponential modelling of 400p-HA showed a greater plateau for the therapeutic group (F test *p* = 0.0021). There was also faster recovery of the ability to perform the B&B tests for the therapeutic group (log-rank test *p* = 0.03).

**Conclusion:**

This study demonstrated the feasibility and potential efficacy of an intensive visual simulation training programme to improve upper limb function in subacute stroke patients. A larger study is needed to confirm these results.

## LAY ABSTRACT

The recovery of poststroke upper-limb motor deficits remains challenging, and improvements in this deficit rely on a combination of intensive and specific rehabilitation techniques. The aim of this study was to demonstrate the feasibility and test the efficacy of a new video-observation training method named intensive visual simulation to improve upper-limb function. Seventeen subacute hemiplegic stroke patients were included in this study; they received a total of 30 sessions of either intensive visual simulation training (movement attempts with corrected visual feedback) for the therapeutic group or sham-intensive visual simulation (movement attempts with uncorrected visual feedback) for the control group. The global hand functioning assessment (400p-HA) was significantly better at 3 and 4 months in the therapeutic group than in the control group, and the recovery curve was better. We conclude that this intensive visual simulation training regimen demonstrates promising benefits for upper-limb motor recovery in hemiplegic patients at a subacute stage.

Stroke is the leading cause of acquired disability in adults in the most developed countries, with up to 80% of stroke patients suffering an upper limb motor deficit (1, 2). After complete hemiplegia, the recovery of a functional hand is achieved by less than 20% of patients despite sustained rehabilitation care (3). New neurorehabilitation technologies based on the understanding of the neural mechanisms of motor control propose diverse and intensive motor training, which is intended to improve this poor outcome (4, 5).

Among these techniques, observational therapies can be defined as a set of motor rehabilitation techniques that rely on the visualization of body movements and activate the mirror neuron system as the main stimulation of the sensorimotor network (6). Mirror therapy (MT) is the princeps technique (7, 8) and is effective for motor recovery in stroke patients (9). However, its use is problematic in patients who have hemineglect, attentional deficit, apraxia, or aphasia. When they are specified, hemineglect or severe aphasia are common exclusion criteria in most of the clinical studies selected in the recent Cochrane review (9), which means that the effectiveness of MT is not clearly established in patients with these associated impairments. The use of videos and a computerized device could overcome some of these difficulties. Action Observation Training therapies includes different set of techniques using video of movements that provide a model of correctly executed movements, either as an external model at a third-person point of view, or at first-person point of view if the screen is positioned in the optical axis between the subject’s eyes and the impaired limb. The first-person point of view provides visual feedback with an embodiment effect comparable to MT, generating kinaesthetic illusions in most patients. If motor imagery alone can improve upper limb motor function (5, 10, 11), synchronous action observation and motor imagery can enhance excitability of the sensorimotor cortex and contribute to motor improvement following stroke (12). We previously developed the first experimental device to successfully enhance cortical motor activity (13) and relieve pain in patients with brachial plexus lesions (14). A library of movements performed with the intact upper limb was video recorded at the first-person point of view and horizontally flipped to serve as visual feedback during training of the impaired limb. This optical and computerized system provides the subject with an immersive 2D image of a correctly executed movement despite the motor deficit. A full device was consequently developed for motor training of the upper limb (IVS™, Dessintey Co., St-Jean-Bonnefonds, France).

In this proof-of-concept study, we tested the feasibility and effectiveness of intensive visuomotor training with the intensive visual simulation (IVS) device to improve upper limb function in a small sample of first-ever subacute stroke patients with an initially complete motor deficit in the upper limb. The objective was to test the effectiveness of the training with corrected visual feedback, where the device normally executes movements (therapeutic group), versus training with uncorrected feedback (control group), where the device actually delivers the movements of the impaired limb. For both groups, this intervention was given in addition to a standard rehabilitation programme.

## METHODS

### Design

A pilot, prospective, randomized parallel-group, examiner-blind trial was also conducted. During a 2-year period, all patients diagnosed with stroke and admitted to the Physical Medicine and Rehabilitation (PMR) inpatient clinic of the University Hospital of Saint-Étienne were screened for possible inclusion in the study. Baseline evaluation was performed prior to randomization. A computer-generated randomization table was generated by a person not involved in the study. Patients were randomly allocated to either the therapeutic (T) or control (C) group based on an assignment schedule and their details were stored in consecutively numbered, sealed envelopes to ensure concealment. Outcome data were collected at baseline (i.e., a few days before intervention), every 2 weeks during the 6 weeks of intervention, and then monthly until 6 months poststroke. Outcome measurements were performed by an independent examiner blinded to group allocation. To maintain blinding, patients were instructed not to discuss any aspects of their intervention with the examiner. The protocol was approved by the ethics committee of the Saint-Étienne University Hospital (No. 200102-JV200125). Patients were provided with written information regarding the study, and written consent was obtained from all patients before participation in the study.

### Patients

The inclusion criteria were male or female, aged 18 to 75 years, able to give consent, right-handed (Edinburgh Inventory > +40), with a complete motor deficit in the left or right upper limb at D0 poststroke (0 MRC score from shoulder to digits), related to a first ischaemic stroke, less than 30 days old at the time of inclusion, and proven by brain imaging. The main exclusion criteria were disabling general illness, a history of neurological or psychiatric disease, a very poor recovery prognosis due to a complete infarct of the middle cerebral artery territory, a visual deficit that could not be compensated for, and cognitive impairment that compromised comprehension or completion of the rehabilitation programme.

### Intervention

Throughout the study, both groups received individualized standard rehabilitation therapy for the upper limb from a physiotherapist (1 hour) or an occupational therapist (1 hour), which was different from the approach used by the therapist who performed the IVS training and who was blinded to the patient’s group assignment. This standard rehabilitation included hands-on therapy, passive and active mobilization, electrophysiotherapy, and task-oriented therapy. This standard therapy was delivered 5 days a week during a part of the day (morning or afternoon) different from the IVS sessions, thus preserving the patient’s fatigue level. This standard treatment continued after the end of the intervention, either in hospital or on an outpatient basis, according to a programme and duration tailored to each patient.

**Fig. 1:**
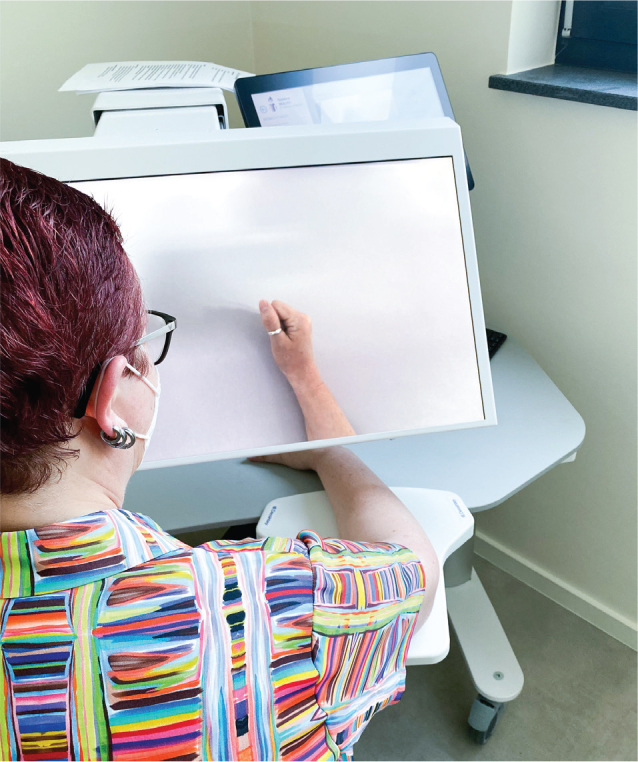
Stroke patient during practice on the IVS device.

Both groups received the intervention with an IVS™ setup (Dessintey Co., France) (Fig. 1). We used a research version of the IVS device, which also allowed direct video capture and display of the impaired limb. The therapeutic group (TG) received upper limb rehabilitation sessions with visual feedback correction (normally executed movements, with horizontally flipped, prerecorded video of the intact limb), whereas the control group (CG) had sessions without visual feedback correction (direct video capture and display of the impaired limb). The training consisted of functional reaching and grasping movements. A set of 10 objects was selected to include 2 examples of 5 types of prehensile postures according to Schlesinger’s classification (cylindrical, tip, palmar, spherical, lateral). Before each movement, a static grey picture with the written name of the next movement to be performed was displayed on the screen for 5 sec, then a cross (preparation cue) for 1 sec, and a video was displayed for approximately 10 sec, either the normally executed movement (horizontally flipped, prerecorded video of the intact limb) for the TG or the direct video capture of the impaired limb for the CG. Patients were asked to perform a volitional movement attempt of the instructed movement during the video display, and for the TG patients were instructed to synchronize this movement attempt with the displayed movement. During each training session, the 10 movements were repeated 20 times, for a total of 200 movements per session. The 2 groups received the same measure of rehabilitation. The therapist remained close to the patient during the training to provide instruction to the patient, ensure optical coherence of the display, eventually assist the patient, and specifically ensure the correct starting position of the patient’s hand. Patients received this training 5 days a week for 6 weeks, for a total of 30 sessions.

### Outcome measures

The main outcome measure was the 400-point Hand Assessment test (400pt-HA) (15–17). This validated test evaluates 57 usual manual activities that test simple distal analytical movements, unimanual grasping and displacement of objects, and the function of both hands (coordinated bimanual movements). By combining analytical and functional dimensions, this test covers a large range of sensitivities regarding motor recovery in the hand.

Additional validated grasping and dexterity tests were also conducted as secondary outcomes: the Box and Block Test (B&B) (18), the Purdue Pegboard Test (Purdue) (19, 20) and the Minnesota Rate of Manipulation Test (Minnesota) (21). To be performed with the impaired limb, these tests require minimal recovery of reaching and grasping capabilities with the impaired limb. These scores are consequently expected to be 0 (or close to 0) at the beginning of the intervention and may remain null in the case of nonfunctional recovery.

An inclusion visit was performed for each patient with a clinical examination, and data regarding associated deficits, such as sensitivity impairment, hemineglect, and praxis disorders, were collected to eventually assess the influence of these factors on therapeutic efficacy. At inclusion, a baseline motor evaluation of the upper limb was performed (400pt-HA, B&B, Minnesota, Purdue). This motor evaluation was repeated every 2 weeks during the 6 weeks of the rehabilitation programme and then monthly until 6 months poststroke. These numerous assessments (8 per patient in the case of complete data), with a relatively long follow-up period (6 months poststroke), were designed to allow temporal modelling (estimate of the recovery curves) in addition to the usual comparative analysis.

### Data analysis

All the data were analysed using Prism 5 software (GraphPad Software, Inc; https://www.graphpad.com/). The Mann‒Whitney *U* test was applied to determine the difference in age between the 2 groups, and Fisher’s exact test was applied for the other characteristics of the patients. Comparisons of test results between the 2 groups were performed with Mann‒Whitney *U* tests. Two-tailed results were considered significant if *p* < 0.05. In addition, one-phase exponential modelling was applied to the recovery curves of 400pt-HA for each group, and the fits of the 2 groups were compared with an extra sum-of-squares F test. Considering that dexterity tests (B&B Minnesota, Purdue) have an optional and delayed recovery (non-0 score), additional time-to-event statistics were performed (time-to-non-0 score), similar to survival curves, with a comparison of the two groups of curves using log-rank (Mantel–Cox) tests.

## RESULTS

### Patients included

Seventeen patients, with a mean age of 63.4 years (SD 7.8), who were suffering a first ischaemic stroke and met the inclusion criteria, were enrolled and randomly assigned to the control group (CG, *n* = 9; mean age 64.2 years, SD 9.6) or the therapeutic group (TG, *n* = 8; mean age 62.6 years, SD 5.7). The main characteristics of the population are described in Table I, and none of these characteristics significantly differed between the groups. There was an overall predominance of right hemiplegia (13 out of 17 patients), which was balanced between the groups.

**Table I:** Main characteristics of the patients

Initials	Sex	Age	Affected limb	Brain lesion
**Control**				
DR01	M	62	R	Left centrum semiovale and thalamus
GA03	M	72	R	Left superficial MCA territory
SC07	M	49	L	Right lenticulo-striate territory
DY12	F	72	R	Left superficial MCA territory
GJ13	F	47	R	Left superficial MCA territory and partial lenticulo-striate territory
RL14	M	70	R	Left lenticulo-striate territory
DM15	F	66	R	Left superficial MCA territory
FG16	F	70	R	Left lenticulo-striate territory
GD17	F	69	L	Right superficial MCA territory
**Therapeutic**				
FJ02	M	58	R	Left superficial MCA territory
RM04	F	57	L	Right internal capsule and centrum semiovale
RE05	M	68	R	Left internal capsule and thalamus
VJ06	M	71	R	Left lenticulo-striate territory
GM08	F	66	L	Right lenticulo-striate territory
PL09	M	59	R	Left internal capsule and centrum semiovale
FF10	M	57	R	Left centrum semiovale
BL11	M	65	R	Left internal capsule

MCA: middle cerebral artery.

### 400pt-HA results

**Fig. 2:**
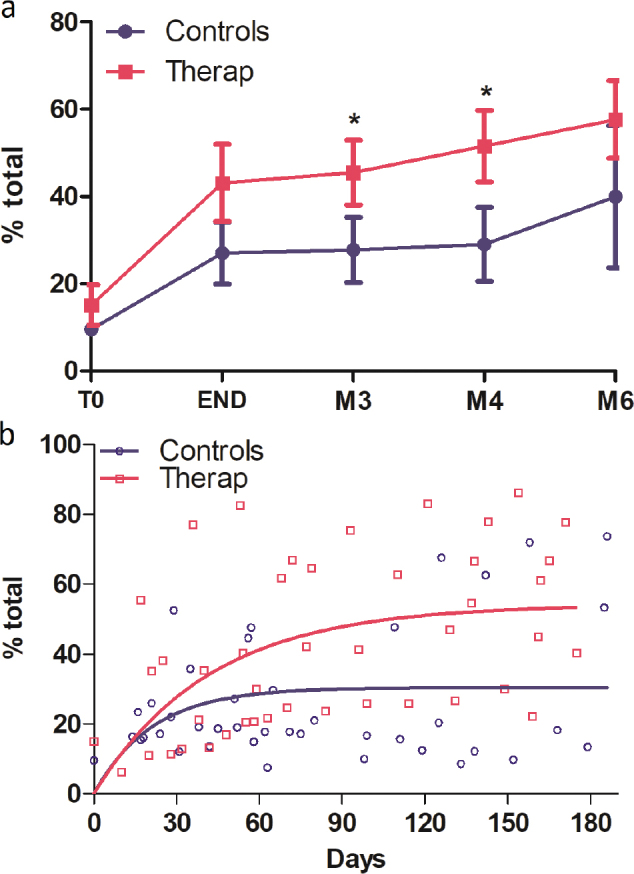
Temporal evolution of the total 400p-HA test, expressed as percentage of recovery of the maximum score, for the therapeutic group (red dots) and the control group (blue dots); (a) mean difference with SD at fixed times: T0, END of intervention, M3, M4 and M6 poststroke. *p < 0.05 Mann–Whitney comparison; (b) one-phase exponential modelling for the therapeutic group (red dots and curve) and the control group (blue dots and curve).

The 400pt-HA test showed a significant improvement from T0 to the end of the intervention (Fig. 2a and Table II) and then a slower improvement until 6 months. The difference in the means between the TG 43.1 and CG 27.0 groups did not reach statistical significance (Mann‒Whitney *U* test *p* = 0.07) at the end of the intervention but became significant during the follow-up period at 3 months (TG 45.5; CG 27.8; *p* = 0.046) or 4 months (TG 51.5; CG 29.9; *p* = 0.046). This difference at 3 and 4 months is superior to the minimal clinically important difference (MCID) of 6. Modelling of the recovery of upper limb function based on all temporal data of patients in each group was performed with a one-phase exponential model, which was selected as the best suitable model for the recovery curve (Fig. 2b). This model differed significantly between the therapeutic and control groups (*p* = 0.0021; extra sum-of-squares F test), with the therapeutic group reaching a higher plateau (Ymax: TG 54.2 with 95% CI [40.3–68]; CG 29.2 with 95% CI [22.6–38.4]).

**Table II:** Details of results for the total 400p-HA test and the 3 dexterity tests over time (T0, END of intervention, M3, M4, and M6) for the therapeutic group and the control group

Test	*Therapeutic group*					*Control group*					
	T0 Mean ± SD	END Mean ± SD	M3 Mean ± SD	M4 Mean ± SD	M6 Mean ± SD	T0 Mean ± SD	END Mean ± SD	M3 Mean ± SD	M4 Mean ± SD	M6 Mean ± SD	MCID Mean ± SD
*400p-HA*	15.1 ± 13.2	43.1 ± 25.0	45.51 ± 20.9	51.6 ± 23.3	40. ± 36.6	9.6 ± 3.2	27.0 ± 21.4	27.8 ± 22.4	29.9 ± 26.5	57.6 ± 25.1	3.3
*Box& Block*	0.7 ± 1.9	2.4 ± 3.7	7.7 ± 10.0	10.1 ± 4.0	2.8 ± 4.1	0.6 ± 1.2	1.9 ± 3.8	4.7 ± 9.5	6.0 ± 10.5	5.0 ± 4.4	5.5/7.8*
*Purdue*	8.3 ± 2.1	13.8 ± 12.9	NA	NA	21.3 ± 12.6	8.9 ± 1.5	12.3 ± 4.1	NA	NA	13.6 ± 8.3	3/6**
*Minnesota*	0	12.6 ± 22.4	NA	NA	22.0 ± 25.6	0	6.4 ± 16.8	NA	NA	10.0 ± 24.5	NA

400p-HA: 400-point Hand Assessment test; NA: non-available; Purdue: Purdue Pegboard test.

* Most affected side/less affected side.

** One hand/both hands; no study available in stroke patients.

### Dexterity tests

**Fig. 3:**
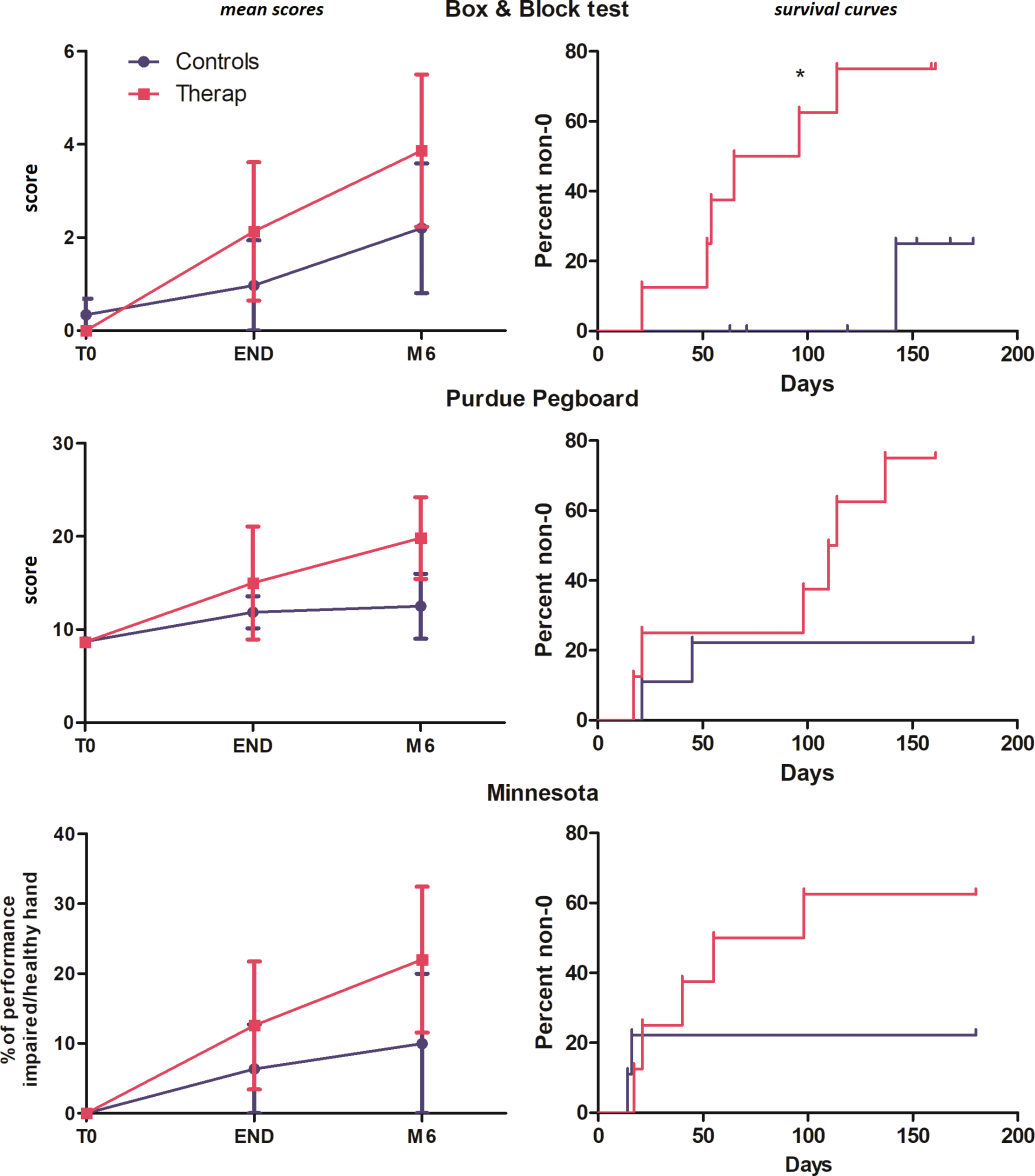
Left column: temporal evolution for the 3 dexterity tests; right column: survival curves showing the % of participants recovering a non-0 score in the 3 dexterity tests.

The mean evolution of the B&B, Purdue and Minnesota tests can be seen in the left column of Fig. 3. The mean values of the 3 tests were consistent in favour of the therapeutic group, although the Mann‒Whitney *U* test did not differ significantly. The cumulative curves of the time-to-non-0 scores can be seen in the right-hand column of Fig. 3. This temporal time-to-event approach allows us to consider the duration of a 0-point interval as a marker of recovery. These curves show a consistently faster recovery of non-0 scores for the 3 tests, with a log-rank (Mantel‒Cox) test indicating significant results for the B&B test (*p* = 0.03); concretely, this finding indicates that the recovery of the ability to transfer at least 1 cube (from one side to the other) with the paretic hand is faster in the therapeutic group than in the control group.

## DISCUSSION

This proof-of-concept study demonstrated the feasibility of innovative motor training based on immersive visual feedback to improve upper limb function in a small population of subacute stroke patients. By using prerecorded videos of movements normally executed with a healthy limb, this technique provides, similar to mirror therapy, a realistic view of the attended movement from a first-person point of view (22). The main advantages of this technique, compared with mirror therapy, are that (*i*) the patient is fully focused on the impaired limb and does not have to produce concomitant movements with the healthy limb and (*ii*) the range of movements can be performed in a large prehensile space and is not limited by the mirror to the hemispace. The simplicity of the task to be performed and the ease of installation on the device probably explain the complete participation of the patients in this intensive training (200 movements per session) at an early subacute stage, less than a month after stroke, as they suffered from a complete motor deficit.

Although conducted on a small population (17 patients), this comparative study provides several positive results supporting the effectiveness of the training with corrected visual feedback, where the device delivers normally executed movements (therapeutic group), versus similar training with uncorrected feedback (control group), on top of a standard rehabilitation programme. The main positive result was obtained with the 400pt Hand Assessment test (400pt-HA), which was defined as the main outcome measure. The mean difference between groups (Mann‒Whitney test) was not significant at the end of the training programme and reached a significant level at 3 and 4 months, respectively around 15 and 20, which is superior to the estimated minimal clinically important difference of 6 (17). This delayed significant difference may, of course, result from the small power of this study but also from the course of motor recovery of the upper limb, which is considered maximal at 3 months, with a plateau after 3 months for most of the patients (3, 23, 24). For this reason, a change in the motor recovery curve due to an early intervention can be statistically detected later in the follow-up period, at the maximum recovery time of approximately 3 months, without questioning the causality of the intervention (5, 24). We may also consider that all the patients received in parallel a standard rehabilitation programme during the intervention period and also during the follow-up period (5 days a week during the inpatient stay, then 2–3 days a week as an outpatient). This standard rehabilitation programme, on top of the spontaneous recovery, also explains part of this continuous improvement after the end of the intervention, and its effect is balanced between groups. At the end of the follow-up period, both groups showed improvement, with a non-significant difference between groups, yet the therapeutic group showed significant improvement earlier. This IVS intervention, thus, may produce an acceleration of recovery, confirming the value of neurofeedback rehabilitation tools for upper limb recovery post-stroke, as supported by literature reviews (5). Because of the sufficient number of repeated measurements available for each patient, approximately 8 measurements were available for a complete follow-up until 6 months, and we were able to construct a recovery curve with the 400pt-HA assessments. This one-phase exponential modelling demonstrated that the therapeutic group reached a significantly greater plateau than the control group. This fitting with a one-phase exponential model is well established for the Fugl–Meyer upper limb test (FM-UL) (3). The 400pt-HA test is a complete and mixed assessment that tests, like the FM-UL test, a panel of elementary movements, and tests graduated functional reaching and grasping movements, like functional tests such as the ARAT. Here, we also showed that the metrological properties of 400pt-HA also respond to one-phase exponential modelling.

The mean scores of the secondary outcome measures, the Box & Block test (B&B) and dexterity test (Purdue, Minnesota), all supported better performance in the therapeutic group, but between-group comparisons at fixed times did not reach the significance threshold. This lack of significance not only results from the small number of patients but also results from a floor effect of the measure, with a mix of delayed ability to perform a non-0 score for some patients and some patients who never gain this ability during the following period. Another statistical approach was then applied to take advantage of the repeated temporal measures available to test the time difference needed to recover the specific ability tested by each test: grasping and displacing a cube for the B&B test, placing a disc in a hole with the paralyzed hand for the placing test of the Minnesota, and placing a small pin in a hole for the Purdue Pegboard Test. We believe that this temporal approach, testing the time to recovery of a specific ability based on robust time-to-event statistics (survival curves), is a simple and sensitive approach that complements the classical performance comparison at fixed times and is very suitable for small-N studies such as this one. Using this temporal approach, we demonstrated that patients in the B&B therapy group had significantly shorter time-to-non-0 scores than patients in the control group. The Minnesota and Purdue tests did not reach the significance threshold. The B&B test, which is recommended in guidelines for post-stroke protocols (24), seems to be the most sensitive test for this temporal approach, probably because it is easier to perform than the Minnesota and Purdue tests, leading to a higher and suitable probability of success.

This study assessed the effectiveness of a video-based visual feedback therapy synchronized with a coherent motor imagery task (Intensive Visual Simulation) in enhancing motor recovery in the upper limb in subacute stroke patients. To our knowledge, 3 other computerized devices have been described more recently: computerized mirror therapy with augmented video feedback (ART system) provided by an avatar of the hand (25, 26); a camera-based mirror visual feedback device (CBMVF), which has also been tested for its ability to enhance upper-limb motor recovery in stroke patients (27); and a video-augmented wearable reflection device (28). Although mirror therapy (MT) has proven to be effective at improving upper-limb motor recovery in stroke patients (9), these 3 devices have also been proposed to overcome some limitations of MT practice with a sagittal mirror, such as posture pressure, lack of engagement, and sustained attention towards the mirror (29), which hinder treatment effects. The efficacy of these 3 devices has also been supported by proof-of-concept studies in stroke patients (26, 27). Compared with these devices, the IVS device combines all the advantages of these 3 devices, such as naturalistic first-person visual feedback in the frontal position, a large working space, a complete focus on the impaired limb, and a large possibility of training analytic and functional exercises.

Complementary to clinical arguments, Ding et al. (27) also conducted neurophysiological EEG recordings to investigate the change in EEG connectivity induced by CBMVF training, suggesting improved local efficiency of communication in the visual, somatosensory, and motor areas induced by the therapy. Additional electrophysiological studies with IVS should also be conducted to highlight some of the innovative properties of the IVS training. An initial one is the comparison between unilateral work with IVS and bilateral work with MT. Unilateral versus bimanual training comprises 2 completely different motor tasks, activating different brain networks, which may be complementary approaches. A second characteristic of the IVS training is the possibility for the patient to control and modulate their motor task practised during the video display of movements: passive observation, motor imagery, or execution attempt. The control of this graded motor task is difficult to achieve with a simple mirror. It modulates the sensorimotor network activity (12) and may be useful to monitor at a patient level, for example with EEG recording, to personalize the training.

This clinical study, like most of the recovery studies on hemiplegic stroke, focused on motor recovery with repetitive and intensive motor training and the improvement of performance on analytic or functional motor tests (30). These research objectives underestimate the role of body representations, which are often altered in hemiplegic patients and need to be evaluated and rehabilitated (31). By providing a repeated visualization of our own body practising movements from the first-person point of view, IVS training may also contribute to restoring altered body representations, and this may be one dimension that underpins the efficacy of this technique. Studies with dedicated designs should be conducted to assess this specific dimension.

In conclusion, this controlled study demonstrated the feasibility and efficacy of a new video observational training method (IVS) for motor recovery in the upper limb in subacute stroke patients. The present data showed that, compared with an intervention with uncorrected visual feedback, intensive training with IVS for 6 weeks improved the global functioning of the impaired limb, as assessed by the 400p-HA test. A faster recovery of a non-0 score for the Box & Block test was also demonstrated. If these results are conclusive for the sample of this proof-of-concept study, the generalization of these results requires a confirmatory study conducted on a larger stroke population.
